# Biocompatibility and Alkaline Phosphatase Activity of Phosphorylated Chitooligosaccharides on the Osteosarcoma MG63 Cell Line

**DOI:** 10.3390/jfb1010003

**Published:** 2010-10-22

**Authors:** Jayachandran Venkatesan, Ratih Pangestuti, Zhong-Ji Qian, BoMi Ryu, Se-Kwon Kim

**Affiliations:** 1 Department of Chemistry, Pukyong National University, Busan 608-737, Korea; E-Mails: venkatjchem@gmail.com (J.V.); pangesturth@yahoo.com (R.P.); ryu.bomi@gmail.com (B.R.); 2 Marine Bioprocess Research Center, Pukyong National University, Busan 608-737, Korea; E-Mail: qianzhongji@hanmail.net (Z.Q.)

**Keywords:** phosphorylated chitooligosaccharides, physiochemical characterization, cytotoxicity, alkaline phosphatase activity

## Abstract

Phosphorylated chitooligosaccharides (P-COS) were prepared using a H_3_PO_4_, P_2_O_5_, Et_3_PO_4_ and hexanol solvent system. The P-COS were characterized by Fourier Transform Infrared Spectroscopy (FT-IR), Thermo gravimetric-Differential Thermal Analyzer (TG-DTA), ^13^C NMR, ^31^P NMR, X-ray diffraction analysis, solubility studies, biocompatibility and Alkaline Phosphatase Activity (ALP). The results reveal that phosphorylation occurred at the C_3_ and C_6_ position of OH groups and the C_2_ position of NH_2_ group. FT-IR confirmed no decomposition in pyranose ring in P-COS even with heating and treatment in acidic conditions. The amorphous nature of P-COS was confirmed by X-ray diffraction analysis. Further, the biocompatibility and alkaline phosphatase activity of P-COS were checked against the osteosarcoma MG63 cell line at different concentrations and no cytotoxicity was observed. After 12 h and 24 h of incubation, the ALP activity of P-COS was higher compared with the control group. These results suggest that P-COS is a biocompatible material and in future P-COS could open up a number of promising pharmaceutical and clinical applications to mankind.

## 1. Introduction

Natural polysaccharides are recommended as bioactive materials, because they possess excellent properties such as biocompatibility, biodegradability, low-toxicity, adsorption properties, *etc*. [1]. Chitosan is a linear polysaccharide consisting of β-(1→4)-2-acetamido-D-glucose and β-(1→4)-2-amino-D-glucose units derived from partial deacetylation of chitin [2,3,4]. There has been a growing interest in chitosan polymer as a promising biomaterial in the pharmaceutical industry in the last three decades. However, the poor solubility of chitosan in water makes it too difficult to be used in food and biomedical applications [5]. Since the discovery of chitosan, several chemical modifications have been tried to improve its solubility and to thus increase its application [6]. Considering this limitation, researchers are now concentrating on conversion of chitosan into oligosaccharides [7]. 

Chitooligosaccharides (COS) have positive charges with D-glucosamine residues; this property enables them to interact with negatively charged polymers, macromolecules and polyanions in an aqueous environment [8,9]. COS are readily soluble in water due to their shorter chain lengths and free amino groups in D-glucosamine units [1]. COS have been shown to possess many biological activities such as antibacterial [7,10,11,12,13], immunoenhancing effect [14], as an antioxidant [15,16], matrix metalloproteinase (MMP) inhibitors [17,18,19], anti-diabetic [20], anti-HIV [21], anti-inflammatory [22], drug delivery [23], *etc*. It is believed that the capability of COS is not only restricted to these activities and that chemical modifications will enhance and open ways for utilization of COS in various further fields [3]. The chemical modifications of COS that have been tried include carboxylation [24], phosphorylation [25] and modification with various lipids such as acryloyl, propionyl, butylyl, isobutylyl, valeryl, isovaleryl, pivaloyl, hexanoyl, octanoyl, decanoyl, lauroyl, myristoyl, palmitoyl, stearoyl, oleoyl, eicosanoyl, docosanoyl, and tetracosanoyl [26]. Compared to natural COS, modified COS are found to be more effective in inhibiting angiotensin converting enzymes [24] and potential inhibitors of calcium phosphate precipitation [25]. The rationale for this is that chemical modification would keep the original physiochemical and biochemical properties of COS and at the same time allow out new additional properties [27]. Among the variety of chemical modifications, phosphorylation is highly used. Several methods have been used for phosphorylation of chitosan that occurs on the surface level, whereas, -H_3_PO_4_/P_2_O_5_/Et_3_PO_4_/hexanol solvent system phosphorylation occurs at the molecular level of chitosan with high yield, high degree of substitution and also a simpler purification process [27,28]. We propose that use of the same solvent system H_3_PO_4_/P_2_O_5_/Et_3_PO_4_/hexanol for the molecular level phosphorylation of COS will increase its potential behavior in pharmaceutical applications.

In this present study, we prepared five different molecular weight P-COS by using the H_3_PO_4_/P_2_O_5_/Et_3_PO_4_/hexanol solvent method and named them as S1, S2, S3, S4 and S5. Compared to the previously reported strategies for COS modification, this method has several advantages, including the mild experimental conditions with no need for purification. Moreover, the cytotoxicity and alkaline phosphatase activity of these five P-COS were examined in human osteoblast-like MG63 cells. These results suggest that in the future, P-COS could open up a number of promising pharmaceutical and clinical applications to mankind. 

## 2. Experimental Section

### 2.1. Materials

Five different molecular weight of COS (<1 kDa, 1–3 kDa, 3–5 kDa, 5–10 kDa and >10 kDa) prepared from crab shells were purchased from Kitto Life Co. (Seoul, Korea). Hexanol, phosphorus pentoxide, phosphoric acid, tri ethyl phosphate, potassium bromide and MTT reagent were obtained from Sigma Chemical Co. (St. Louis, MO, USA). Dulbecco’s Modified Eagle’s Medium (DMEM), Trypsin-EDTA, penicillin/streptomycin, fetal bovine serum (FBS) and other materials required for culturing cells were purchased from Gibco BRL, Life Technologies (USA). 

### 2.2. Synthesis of P-COS

Five kinds of P-COS were prepared, according to a previously reported method with slight modification [27]. 1.0 g of COS powders were mixed with 10 ml hexanol and a mixture of P_2_O_5_ (10 g), H_3_PO_4_ (5 mL), Et_3_PO_4_ (5 mL) was added to the COS solution. Then, the reaction mixture was stirred continuously for 72 h at 35 °C. After 72 h, an excess amount of methanol was poured into the reaction mixture. The brown color solid product was filtered and then washed with an excess amount of methanol. The products were dissolved with double distilled water and then freezed at −80 °C for 5 h and lyophilized. The dried products were kept in the desiccator for further analysis. 

### 2.3. Characterization

For Thermo gravimetric-differential thermal analysis, COS and P-COS powders were uploaded into a Perkin-Elmer (USA) Pyris 1 TGA analyzer. Samples were scanned in a temperature range from 50 to 700 °C at a constant rate of 10 °C min^−1^ with continuous nitrogen flow and DTA curves were obtained. The dried samples were mechanically blended with 100 mg of KBr. The mixture was compacted using an infrared spectroscopy hydraulic press at a pressure of 8 tons for 60 s. The spectra of samples in the form of KBr disks were obtained using a FT-IR spectrometer (Perkin Elmer spectrum GX, Beaconsfield Bucks, England) with frequency range 400 cm^−1^ to 4,000 cm^−1^. For X-ray diffraction analysis, the P-COS powders was analyzed through PHILIPS (Netherland), X’Pert-MPD diffractometer, at 30 kV and 25 mA, and Cu-Kα radiation (1.5405A°) range 5 to 80° angle at a rate of 2 °C for 0.1°. The ^13^C and ^31^P NMR spectra of the P-COS were recorded in D_2_O on a JNM-ECP-400 with a JEOL-Japan, 400 MHz spectrometer. Human osteoblast-like MG63 cells were obtained from the American Type Culture Collection (Manassas, VA). MG63 cells were grown as previously described [29]. In brief, cells were grown in 75 cm^2^ plastic tissue culture flasks (Falcon) in DMEM medium (Gibco) supplemented with 10% fetal bovine serum, 100 units/mL penicillin and 100 μg/mL streptomycin. Cells were maintained at 37 °C in a 95% air, 5% CO_2_ atmosphere. The culture media was changed three times a week. The cell viability of MG63 cells were assessed via 3-(4,5-dimethyl-2-yl)-2,5-diphenyltetrazolium bromide (MTT) method. The cells were plated at a density of 1 × 10^4^ cells/well in 96 well plates. On the following day, the cells were treated with different concentrations of P-COS and incubated for 24 h. The MTT assay relies primarily on mitochondrial metabolic capacity of viable cells and reflects the intracellular redox state. After incubation, cells were treated with the MTT (Sigma, USA) solution (final concentration, 1 mg/ml) for 4 h. The medium was removed and 100 µL of DMSO was added to each well. The formazan dye crystals were solubilized for 15 min and relative cell viability were determined by measuring the absorbance at 570 nm using a GENios microplate reader (Tecan Austria GmbH, Austria). For estimation of ALPase activity, osteoblast-like cell were grown to confluence in 24 well plates with DMEM containing 5% FBS. The medium was replaced with osteogenic DMEM supplemented with phosphorylated chitooligosaccharides and cells were incubated for 12 h and 24 h. After the incubation, the cells were rinsed with PBS buffer, homogenized in 25 mM carbonate buffer (pH 10.3) containing 0.1% Triton X-100. Next, the cellular activity was measured by incubating for 30 minutes at 37 °C in 250 mM carbonate buffer containing 1.5 mM MgCl_2_ and 15 mM para-Nitro Phenyl Phosphate (p-NPP). In the presence of ALP, p-NPP is transformed to p-nitro phenol and inorganic phosphate. The ALP activity of the P-COS was determined by measuring the absorbance at 405 nm in a spectrophotometer.

## 3. Results and Discussion

### 3.1. General description

The color of the P-COS (<1 kDa—S1) sample was found to be pale yellow as compared to other molecular weight P-COS (1–3 kDa—S2, 3–5 kDa—S3, 5–10 kDa—S4, and >10 kDa—S5). As the molecular weight increases, the color of the product deepens as brownish yellow color. X-ray diffraction result reveals that P-COS and COS appeared as amorphous in nature. 

### 3.2. Solubility of P-COS

Solubility is the most important factor in pharmaceutical drug development. Solubilities of P-COS are shown in Table 1. In this case, we have used 11 type of solvents, P-COS is easily soluble in water, hydrochloric acid, dilute acetic acid and sodium hydroxide. However, in the case of organic solvent the solubility of P-COS is limited. Based on its solubility in common solvents, we can confirm that P-COS is a promising candidate for pharmaceutical drug development.

**Table 1:** Solubility of phosphorylated chitooligosaccharides (P-COS).

Solvent	Chitooligosaccharides	P-Chitooligosaccharides
H2O	Soluble	Soluble
Acetic acid (1%)	Soluble	Soluble
NaOH (1%)	Soluble	Soluble
HCl (1%)	Soluble	Soluble
Dimethyl sulfoxide	Soluble	Soluble
Dimethyl acetamide	Insoluble	Insoluble
Pyridine	Swelling	Swelling
Dimethyl formamide	Insoluble	Insoluble
Ethanol	Insoluble	Insoluble
Acetone	Insoluble	Insoluble
Chloroform	Insoluble	Insoluble

### 3.3. Stretching frequency P-COS

**Figure 1:**
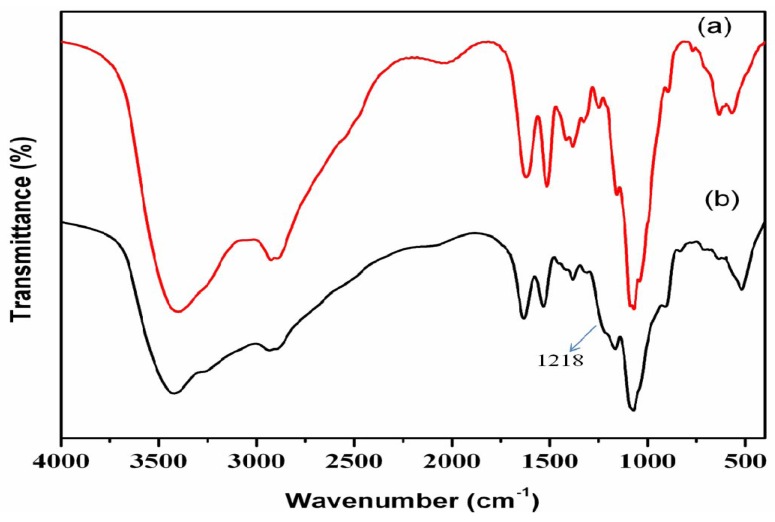
FT-IR spectra of (**a**) Chitooligosaccharide (COS; red line) and (**b**) Phosphorylated chitooligosaccharide (P-COS; black line).

The spectra of unmodified COS and P-COS are shown in Figure 1. The spectrum of unmodified COS showed characteristic peaks of amide I at 1,620 cm^−1^ and amide II at 1,514 cm^−1^. The broad peak observed at 3200–3500 cm^−1^ is due to the overlapping of different vibrations corresponding to the OH and amine groups. The other peaks at 2,890 cm^−1^ and 1,380 cm^−1^ were assigned to CH stretching and CH_3_ symmetric deformations. The shoulder peaks was observed for P-COS at 1,218 cm^−1^, which can be attributed to the P=O asymmetric stretching from phosphates. This clearly confirmed that phosphorylation occured in the COS moiety. The hydroxyl group absorption in COS was not observed at 1,320 cm^−1^. This confirmed that phosphorylation occured at all the OH groups of COS [28,30].

There was no change in the absorption at 1,147 cm^−1^ which indicated that there is no decomposition in the pyranose ring of P-COS due to heating and acidic conditions. 

### 3.4. Thermal stability of P-COS

**Figure 2:**
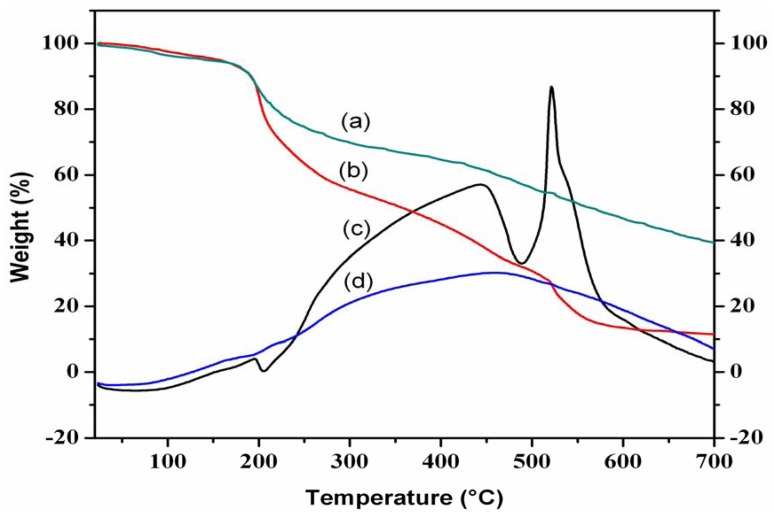
TG-DTA curve of (**a**) TG-chitooligosaccharide (**b**) TG-P-chitooligosaccharide (**c**) DTA-Chitooligosaccharide (**d**) TG-P chitooligosaccharide.

The TG-DTA curves of COS and P-COS are shown in Figure 2. Here, no significant differences were found between COS and P-COS. The TGA curve of COS showed two different types of peaks at 100 °C and 200 °C. The first weight loss attributed to loss of water molecule. The second stage weight loss observed at 200 °C might correspond to degradation of pyranose ring of COS. In the case of P-COS, no weight loss was observed at 100 °C, but weight loss was observed at 200 °C, like for COS. P-COS loses weight around 80% then compared to unmodified COS (60%). This might corresponds to the removal of phosphorylated groups in P-COS.

### 3.5. ^13^C NMR spectra of P-chitooligosaccharide

**Figure 3:**
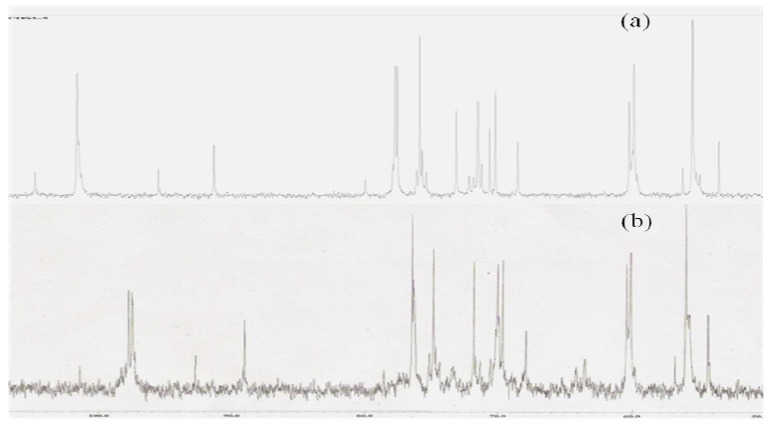
.^13^C NMR spectra of (**a**) chitooligosaccharide (COS), and (**b**) phosphorylated chitooligosaccharide (P-COS).

Figure 3(a and b) depicts the ^13^C NMR spectra of COS (3–5 kDa) and P-COS (3–5 kDa—S3), respectively. All the peaks are well separated and correspond to each carbon atom. No other additional peaks were observed corresponding to aldehydic and ketonic groups. This revealed that no decomposition occurred in pyranose ring. 98.4, 56.0, 70.8, 76.4, 74.8 and 60.2 ppm refers to the C1, C2, C3, C4, C5 and C6 positions of COS (3–5 kDa), respectively. Whereas, for P-COS the peaks were different from raw 3–5 kDa chitooligosaccharide. The chemical shift of the C6 position moves from 60.2 to 60.4 ppm and for C-3 from 70.8 to 70.0, indicating the substitution of phosphate group. These results indicated that the phosphorylation reaction occurred in chitooligosaccharides.

### 3.6. ^31^P NMR spectra of P-chitooligosaccharide

**Figure 4:**
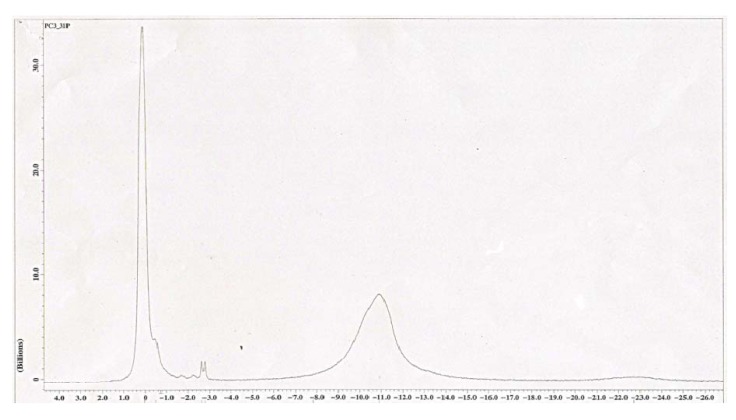
^31^P NMR spectra of S3—POS.

^31^P NMR technique is a very good tool to confirm phosphorylated compounds. The ^31^P spectra of P-COS is shown in Figure 4. The different degrees of substitution and position of phosphorylation in COS could be easily identified from the chemical shift of ^31^P NMR spectra. Based on ^31^P NMR results, formation of the phosphate group on COS is considerable and the primary hydroxyl group is more reactive than the secondary hydroxyl groups. In addition, the appearance of a small peak around −0.5 indicated that another kind of group in COS was also phosphorylated. According to Wang *et al*., if the degree of substitution is low we get a single peak, whereas, if the degree of substitution is more we get three peaks. This suggested that, the C_6_ position of hydroxyl group of COS is phosphorylated. Another two peaks at −10.8 and −2.7 might be due to phosphorylation at other positions like C_2_ and C_3_. The low intensity peak observed at −2.7 indicated that one kind of amino group is phosphorylated.

### 3.7. Biocompatibility of P-COS in an osteosarcoma–cell line

**Figure 5:**
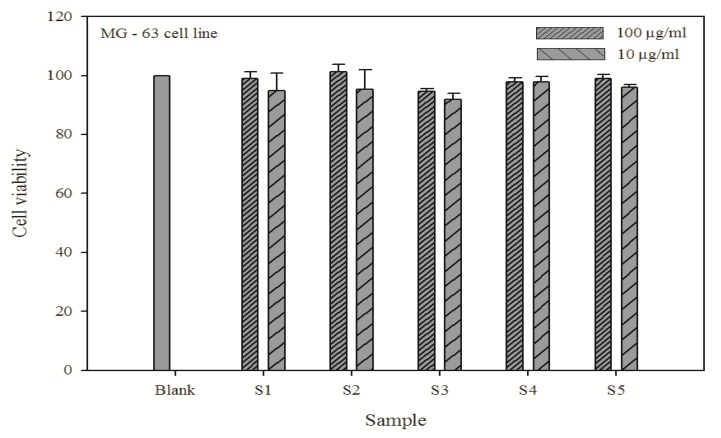
Cytotoxicity effects of the phosphorylated chitooligosaccharides in the MG63 cell line. The error bar indicates the standard variation of three parallel experiments.

The biocompatibility of P-COS was tested against MG63 cells by MTT assay. The results are shown in Figure 5 and suggest that chemically modified COS has a potential role in growth of the MG63 cell line. No cytotoxicity was observed with different molecular weights of P-COS at the concentration of 100 µg/mL and 10 µg/mL. Moreover, with lower molecular weight P-COS, the cells proliferated more than with higher molecular weight P-COS; thus the phosphorylated group may induce cell proliferation. 

### 3.8. ALP activity of phosphorylated chitooligosaccharides

**Figure 6:**
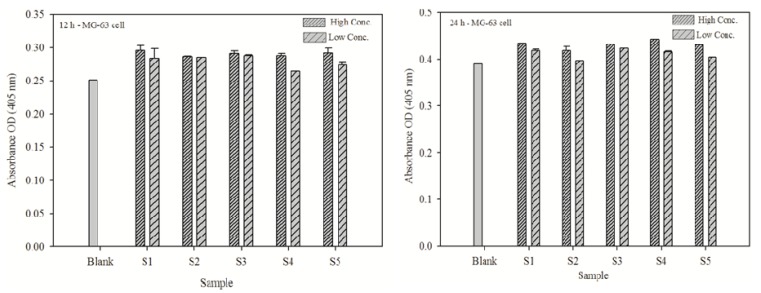
Alkaline phosphatase activities at 12 and 24 h after treatment of osteoblast-like MG63cells with different molecular weight phosphorylated chitooligosaccharides (low conc. 10 μg/mL, high conc. 100 μg/mL). The error bars indicate the standard variation of three parallel experiments.

ALP activity measurement was carried out to analyze the effect of phosphorylated chitooligosaccharides on the osteoblast-like MG63 cell line. The measured ALP activity is shown in Figure 6 (low conc. 10 µg/mL, high conc. 100 µg/mL). ALP level was significantly increased in the presence of phosphorylated chitooligosaccharides compared to control. However, no significant difference was found between the presence of the different molecular weights phosphorylated chitooligosaccharides. At 12 h ALP activity of phosphorylated chitooligosaccharides, the absorbance at 405 nm was found to be around 0.28, whereas at 24 h, it was found to increase to twice its value at 12 h (>0.45).

## 4. Conclusions

In the present study, five different molecular weight P-COS were prepared by H_3_PO_4_/P_2_O_5_/Et_3_PO_4_/hexanol solvent system. ^31^P NMR results suggested that phosphorylation occurs at all the reactive positions of COS (at the C_2_ amino and OH groups at C_3_ and C_6_ positions). There is no decomposition in P-COS due to the heating and acidic condition, the pyranose ring is very stable and is confirmed by infrared spectroscopy. The results suggest that the P-COS lead to no cytotoxicity and an increase in the ALP activity on MG63 cell line. It is proposed that low molecular weight P-COS could be useful for various biomedical applications.
